# The Dynamic Change in the Reliability Function Level in a Selected Fire Alarm System during a Fire

**DOI:** 10.3390/s24134054

**Published:** 2024-06-21

**Authors:** Jacek Paś, Tomasz Klimczak, Adam Rosiński, Marek Stawowy, Stanisław Duer, Marta Harničárová

**Affiliations:** 1 Division of Electronic Systems Exploitations, Institute of Electronic Systems, Faculty of Electronics, Military University of Technology, 2 Gen. S. Kaliski St, 00-908 Warsaw, Poland; jacek.pas@wat.edu.pl; 2 Department of Building Safety, Fire University, 52/54 J. Słowackiego St., 01-629 Warsaw, Poland; tklimczak@apoz.edu.pl; 3 Department of Air Transport Engineering and Teleinformatics, Faculty of Transport, Warsaw University of Technology, 75 Koszykowa St, 00-662 Warsaw, Poland; adam.rosinski@pw.edu.pl; 4 Department of Energy, Faculty of Mechanical Engineering, Technical University of Koszalin, 15–17 Raclawicka St., 75-620 Koszalin, Poland; stanislaw.duer@tu.koszalin.pl; 5 Department of Mechanical Engineering, Faculty of Technology, Institute of Technology and Business in České Budějovice, Okružní 10, 370 01 České Budějovice, Czech Republic; harnicarova@mail.vstecb.cz; 6 Institute of Electrical Engineering, Automation, Informatics and Physics, Faculty of Engineering, Slovak University of Agriculture in Nitra, Tr. A. Hlinku 2, 949 76 Nitra, Slovakia

**Keywords:** reliability, fire alarm system, fire scenario, power supply, system

## Abstract

This article discusses fundamental issues associated with the functional reliability of selected fire alarm systems (FASs) in operation during building fires. FASs operate under diverse external or internal natural environmental conditions, and the operational process of FAS should take into account the impacts of physical phenomena that occur during fires. Their operation is associated with the constant provision of reliability. FAS designers should also consider the system’s reliability when developing fire control matrices, tables, algorithms, or scenarios. All functions arising from an FAS control matrix should be implemented with a permissible reliability level, R_DPN_(t), prior to, as well as during, a fire. This should be assigned to the controls saved in the fire alarm control unit (FCP). This article presents the process by which high temperatures generated during a fire impact the reliability of FAS functioning. It was developed considering selected critical paths for a specific scenario and the control matrix for an FAS. Such assumptions make it possible to determine the impact of various temperatures generated during a fire on the reliability of an FAS. To this end, the authors reviewed that the waveform of the R(t) function changes for a given FAS over time, Δt, and then determined the fitness paths. The critical paths are located within the fire detection and suppression activation process, using FAS or fixed extinguishing devices (FEDs), and the paths were modeled with acceptable and unacceptable technical states. The last section of this article defines a model and graph for the operational process of a selected FAS, the analysis of which enables conclusions to be drawn that can be employed in the design and implementation stages.

## 1. Introduction

Electronic security systems (ESSs) are intended to provide security in buildings and over vast areas [1,2]. FASs play a particular role in providing safety [3,4]. In most cases that require ensuring a specific safety level, buildings or entire areas decommissioned from public use are often classified as so-called ‘state critical infrastructure’ (SCI) [2,3,4]. The possibilities offered by the integration of individual ESSs used during fire situations and in fire-fighting operations are applied to ensure the specific operational safety of these facilities located within SCI areas [5,6,7]. The most common ones employed within the process of integrating ESS within SCI-classified facilities and areas include intrusion detection systems (IDSs), access control systems (ACSs), closed-circuit television (CCTV), FASs, and audio warning systems (AWSs) [8,9,10]. In such cases, all ESSs in operation within a vast SCI area provide active internal and external protection. Pursuant to valid regulations applicable in the EU or a given country, FASs are intended for the detection of fire phenomena and hazards. FASs are always integrated with AWSs to ensure appropriate and permissible available safe egress times (ASETs) [11,12,13,14]. A significant problem is the functioning of FAS during the fire process at a specific time, Δt. The time interval Δt is a function of many variables, but in the case of a fire, the decisive parameter is always the temperature. The authors of this article conducted operational tests on ten FASs. They determined the degree of damage and required restoration and performed a computer simulation of the impacts of temperature on the reliability of the FAS if used during a fire. The obtained results and FAS reliability calculations for the case of a fire are necessary to design the entire system properly.

### 1.1. Application of Integrated FAS with Varying Functional Structures within SCI-Classified Facilities and Vast Areas

**Figure 1:**
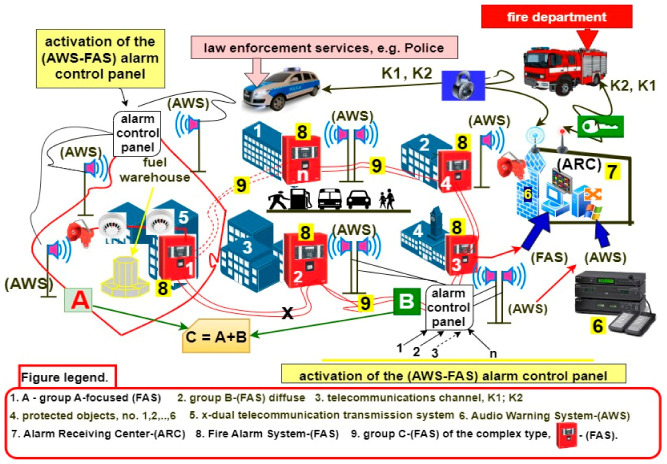
Simplified organizational diagram of two integrated ESS–FAS and an AWS applied within a vast critical infrastructure area, taking into account a fuel depot monitored by a concentrated FAS and a distributed AWS. Individual FACUs (1, 2, …, *n*) are connected with double lines, and the designations are explained in the figure legend.

The most important ESSs that were in operation in SCI facilities, pursuant to applicable acts and regulations, include the following two systems: FAS and AWS [11,15,16]. FASs are always integrated with AWSs in terms of hardware and information. Other security systems that operate in SCI areas and buildings only receive FAS information on the technical state. This is an alarm signal associated with a fire hazard. All FASs and AWSs in operation can be divided, according to the followed functional and technical structures, into three groups. The organization of a given group, including the technical facility’s functional structure, is always a function of the area (volume) of the protected facilities. The given group determines the method of connecting detection loops and circuits that contain the sensors. This also includes the method of connecting AWS loudspeaker transmission lines to the FCP [1,3,11] (Figure 1). The simplest FAS structures include a so-called ‘concentrated system’ for securing small facilities. Then, the start and end of the detection lines and circuits (i.e., inputs and outputs) are always located in the FCP. So-called ‘distributed’ FASs are employed in large-sized facilities. A system includes several or even several hundred FACUs with hooked-up sensors for detecting fire characteristic values (FCVs) [17,18,19]. A structure that combines the two groups referred to above can be classified as an ‘FAS complex structure’. This technical structure type is employed in the case of SCI areas or fire monitoring of important building(s) [14,20,21]. Figure 1 shows the simplified organization of two integrated ESS–FAS and AWS that operated within an SCI area [1,5]. The method of laying and “routing” signal cables must also consider the existing environment and areas of strong electromagnetic interference [1,22]. A fiber optic cable is employed to transmit signals from the FCP or ACU [23,24]. The information on the technical states of the FAS is sent to an alarm-receiving center (ARC). The alarm signal (fire hazard) itself is sent via two independent channels to the State Fire Service (PSP). Evacuation and broadcast messages related to the existing threat are generated automatically [2,8]. The correct operation of all ESSs functioning in SCI areas and buildings depends on the power supply’s reliability. This is an important issue, which is approached separately under ESS functional reliability [25,26,27].

### 1.2. Fire Scenario and Its Implementation during Monitoring and Alarming—Ensuring the Reliability of Controls during a Fire

The definition and general concepts associated with developing a fire scenario were introduced by the provision of the Regulation of the Minister of Internal Affairs and Administration (MSWiA) of 5 August 2023. Art. 5 and 6.1 of the aforementioned regulation specify the requirements and grounds for a fire protection expert’s employment. Pursuant to this regulation, these particularly include the following activities: “selection of fire-fighting equipment, as well as other fire-safety systems and devices, together with specifying their application range and purpose”.

**Figure 2:**
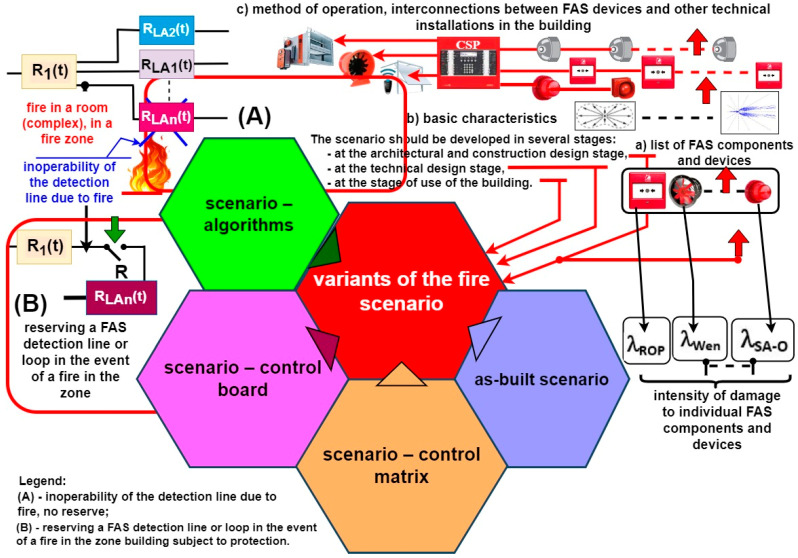
Development and implementation stages of a building fire scenario, in accordance with applicable regulations—FAS, FED, AWS. R_1_(t)—FCP reliability, R_LA1_(t), …, R_LAn_(t)—detection loop circuit, λ_ROP_, λ_Wen_, …, λ_SA-O_—FAS element failure intensity.

Art. 5 requires consultation related to design solutions in terms of evaluating their conformity with fire protection requirements; exchange of comments and statements regarding the designed fire protection technical measures; development of a fire scenario for a building or part thereof that constitutes a separate fire zone, wherein the application of a fire alarm system, fixed extinguishing devices, smoke removal devices, or smoke prevention devices have been planned. According to this regulation, a fire scenario includes a description of potential events occurring during a fire concerning a given location or the entire area of action [14,28,29]. The location of this phenomenon is analyzed in a fire scenario. This involves determining the functional procedures for fire protection equipment (FAS, FED, etc.) installed within a facility. Hence, a fire scenario plan is an agreement on organizational activities required and necessary for the designed protections to function properly. Construction design development practice indicates different levels of detail; therefore, a given fire scenario should always be developed in multiple stages and should apply to different stages of construction and use, i.e., architectural and building, technical and detailed designs (before building operation) (Figure 2). It is important not to forget to introduce follow-up changes in a given fire scenario. In particular, this should be implemented at the as-built stage [10,30,31].

The most crucial task of a fire scenario is to determine such procedures for each event exhibiting the features of a fire occurring within a given building to result in automatic or manual triggering of AWS operation and evacuation announcement procedures related to the people residing therein. The development of a fire scenario should take into account the failure intensity of elements and devices making up this facility, as illustrated in Figure 2 (λ_ROP_, λ_Wen_, …,λ_SA-O_). The manufacturers of FAS and AWS elements do not provide such information; therefore, developing proper operation process reliability structures is a major issue. In his/her fire scenario, a designer must include available technical solutions to enhance R(t) reliability, i.e., redundancy, provisioning, or fail-safe solutions.

This is particularly important in the event of building fire, since in this period (even if limited), these devices must operate and enable proper fire-fighting process implementation. Figure 2 illustrates only one technical solution improving (FAS) reliability in the form of R provisioning of the entire monitoring line R_LAn_(t). Fire-related transition processes, especially temperature, induce a dynamic (transient or permanent) change in ESS reliability structures that is often difficult to determine [6,32,33]. The designer should develop such a reliability structure, so that a fire breaking out in a room or fire zone does not result in the occurrence of a so-called ‘critical unfitness’ of the entire technical object monitoring the facility.

### 1.3. FAS and GES Power Supply during a Fire

**Figure 3:**
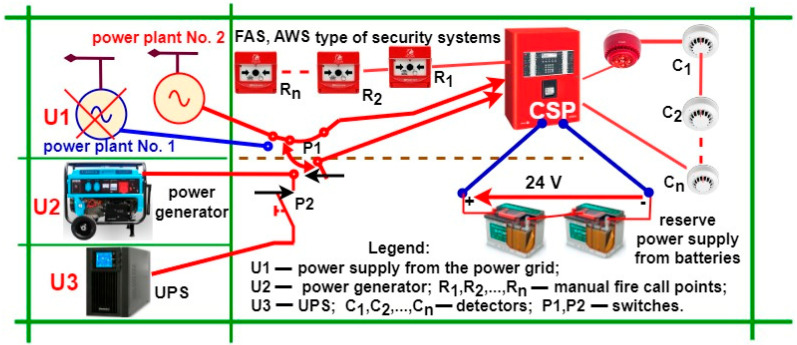
Simplified power supply diagram for FSTMs (fire safety technical measures) over time t_a_, t_d_, and t_p_.

FAS and GES power supply within SCI areas is important during a fire and in fire-fighting operations, whether via Fixed Extinguishing Devices (FEDs) or GESs. When implementing its operational task, an AWS requires power supply continuity. These electricity supply processes must be implemented with the highest possible power supply reliability [34,35,36]. The ESS power supply should be executed from two independent power lines, with additionally provided redundancy in the form of a generator set (GS) and a battery bank connected to an FCP. Portable sensor trip indicators located outside of rooms illuminate (indicate) the evacuation and fire-fighting operation route [2,8]. Figure 3 shows the FAS power supply diagram. It should be implemented over the time of t_d_—monitoring, t_a_—alarming, and t_p_—fire.

## 2. Literature Review

FAS, AWS, FED, GES, etc., operating in SCI areas and buildings must satisfy one crucial requirement. This requirement is to achieve reliability throughout the entire operation period, i.e., t_d_, t_a_, i, t_p_. This means guaranteeing appropriate electricity quality and supply continuity [3,37,38]. ESS functional reliability must be ensured throughout the entire operation process and be taken into account in the control matrix. This includes specific connections, controls, and technical structures. All as-built changes or ESS modernization should not impact (much less deteriorate) the initial reliability R_0_(t) at the time of commissioning of these technical objects [39,40,41].

### 2.1. Review of the Source Literature on ESS Diagnostics

The issue associated with ESS operation that requires attention is its diagnostic process. ESS diagnostics should be automatic, with a remote control option. Also, this must be concurrent with the system’s working and real-time signals [42,43,44]. The diagnostic process should enable a credible assessment of the technical states of all elements making up an ESS [45,46,47]. The surveillance process should also be implemented in the course of a fire hazard—devices operating under adverse environmental conditions. Important ESS-related issues include the visualization of diagnostic events and the transmission of diagnostic information to surveillance sites (local or remote). Any diagnostic information sent or employed locally must be encrypted and modulated by an interference-resistant signal. The authors of the paper believe that, in addition to current diagnostic information related to ESS, a system should also develop predictions of the future technical state [1,48,49]. Contemporarily operated ESSs lack such technical solutions [50,51,52,53].

### 2.2. Overview of Issues Related to Processes Associated with the Occurrence of Natural Environment Interference

Another group of issues encountered in the course of ESS operation includes those which are associated with the occurrence of intentional or unintentional environmental interference (stationary or mobile) [54,55,56]. The authors believe that all types of phenomena adverse to operation processes, so-called ‘mechanical interference’, wall oscillations and vibrations (building penetrations), dust, humidity, pressure, temperature changes, and electromagnetic field interference should be taken into account when choosing and positioning fire event sensors (detectors) [28,57,58,59]. The presence of these unfavorable phenomena within the operation process should already be taken into account at the ESS design stage. According to the article’s authors and available source literature studies, there are no standards and legal requirements in this regard [60,61,62,63]. Proceeding with the design process requires prior testing of the electroclimate, including vibrations and noise [64,65,66,67]. Such solutions (environmental measurements and assessment) also mean a lower probability of ESS false alarms [68,69,70,71].

### 2.3. Overview of Operational Issues Related to Power Supply

ESS power supply in buildings requires undertaking specific actions to ensure a certain quality and reliability level [72,73]. According to the authors, this is most often achieved through employing the redundancy principle [74,75,76]. Redundancy is achieved by the provision of a UPS power generator, two independent power lines, and an accumulator battery being assigned directly to a given system [73,77,78]. Implementing an operational task requires incurring additional financial expenditure associated with the proper organization of the power supply process [79,80]. This also includes organizing and equipping service technician groups, local or remote, with a so-called “on-site” spare parts storage [81,82]. The very signal transmission process also requires attention [2,83,84,85]. The power supply quality, i.e., decays, dips, network harmonics content, etc., constitutes an important issue related to electricity supply [48,86,87]. ESS power supplies should be equipped with elements protecting the entire energy transmission chain [88,89,90]. The authors, however, were not able to not come upon data that would take into account the interference in the natural environment [1,91,92].

### 2.4. Overview of Issues Associated with the Operation of ESS Sensors—Detectors

Sensors responding to different and variable physical phenomena are responsible for detecting a hazard, namely, an FCV change [3,19,93]. These, whether in a detection loop or circuit that is significantly remote from an FCP, determine the possibility of detecting a hazard with a specific probability value [94,95]. The primary signal, brought into being in the detector, greatly impacts false alarm signals generated throughout the entire process [3,95,96,97]. The authors believe that such signals should be primarily mitigated at the source itself. This can be achieved through the application of modern, e.g., dual multisensors (smoke, temperature, or electromagnetic radiation) [98,99,100]. This also includes the implementation of the hazard detection process in detectors through the application of artificial neural networks [101,102,103,104]. Sensors used within ESS monitoring SCI buildings and areas should exhibit appropriate resistance, durability, and susceptibility to occurring interference. This should be taken into account at the process design and modernization stages [3,53,79,105,106].

## 3. The Application of Fire Alarm and Gas Extinguishing Systems in Critical Infrastructure Buildings and Over a Vast Area

**Figure 4:**
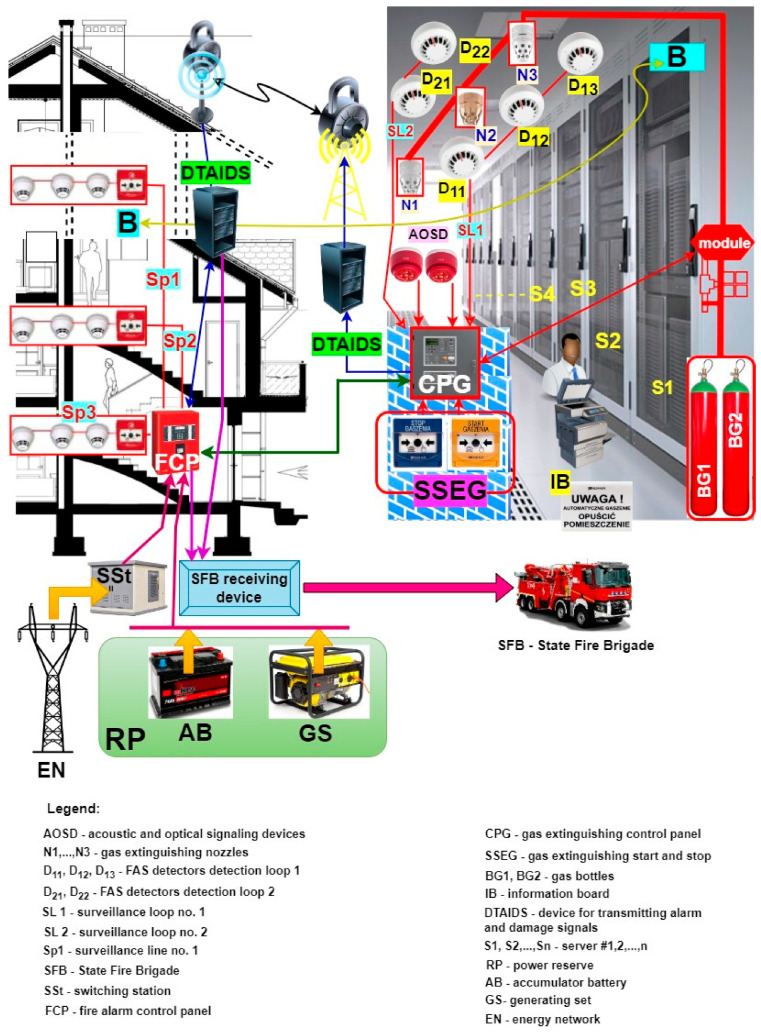
Simplified diagram illustrating the application of FAS and GES in a building with a single B-type room—server room supervised by a separate GES (gas extinguishing system). For the sake of clarity, Figure 4 shows a simplified system organization and technical structure, and the power supply method employing a power grid and redundant sources. The designations in the figure are explained in the key.

FAS and GES are employed to ensure the safety of life, health, and gathered property, as well as to protect the natural environment [3,107,108]. Various technical structures of such systems are used to this end. Three primary structures can be distinguished—focused, distributed, and mixed [40,109,110,111]. Figure 4 shows a focused FAS structure supplemented by an additional GES. A computer server room is located in the room of the building (B), which is monitored by a GES because of the computer systems it houses. In the figure, GES is triggered automatically by the following detectors: D_11_, D_12_, D_13_—circuit SL1 and D_21_, D_22_—circuit SL2. In light of using gas to extinguish a server room fire and reduce the probability of a false alarm, a coincidental or dual-sensor alarm triggering method from different detection lines SL1 and SL2 was employed (e.g., sensors D_12_—circuit SL1 and D_21_—circuit SL2) [9,112]. GES offers a manual method of starting and stopping the entire process through the START and STOP buttons (SSEG) located within the monitored room (STOP) and next to the room entrance (START). The signaling part of the entire gas extinguishing process within a given room is implemented through acoustic and optical signaling devices (AOSDs) and illuminated warning boards placed in spots visible to people using the server room, both outside and inside the fire-fighting zone. The entire GES is controlled by CPG and an electronic module connected to the extinguishing system [3,113,114]. The GES in Figure 4 utilizes two cylinders with compressed inert gas (BG1, BG2) and a system for distributing compressed gas to outlet nozzles (N1, N2, N3) that are additionally equipped with acoustic pressure dampers. To ensure an appropriate level of GES activation reliability in the room of the building, the system includes a gas extinguishing system control panel equipped with a device for transmitting alarms and damage signals (DTAIDS) [40,115,116]. CPG autonomously triggers the gas extinguishing system and controls the entire fire-fighting system and two independent alarm signal transmission channels—wireless and hard-wired signals. For the sake of clarity, Figure 4 illustrates the wireless system only. 

An FAS or GES transmitter automatically sends the alarm signal information to the SFB via the monitoring system. Information on the technical states of the aforementioned systems (alarm, monitoring, or failure) is sent to the ARC (all states), while the SFB only receives information on the alarm state. In the case of the presented solution (Figure 4), if a CPG fails, the process of controlling and triggering the entire procedure may be individually undertaken by the FAS FCP [1,117,118]. For this purpose, the FCP has been equipped with an extinguishing control module that is able to implement all the functionalities of a CPG. In addition, cabling has been laid between individual devices controlled within the GES and the FCP. If necessary, the cables can be connected by the service group to the extinguishing control module that takes over CPG functions. To limit the time associated with deactivating the protection, the cabling is pre-prepared and routed to devices, but not connected to them. The FCP is additionally certified for conformity with the PN-EN 12904:2006 standard [119]. Moreover, information from individual detection lines SL1, SL2 on the fire hazard is sent to the FCP, where an extra module responsible for implementing the gas extinguishing process only is installed. The FAS includes three detection loops (SP1, SP2, SP3) that monitor individual floors and passageways, and are equipped with sensors and manual call points (MCPs). What is more, the FAS has been fitted with a DTAIDS transceiver, which receives technical state signals from the GES. An additional DTAIDS is organized for the purposes of the FAS alone [40,120,121]. After the alarm is acknowledged by the local operators in the building, the alarm signal is sent from this transmitter to the SFB. 

All information on the FAS and GES technical states is encrypted and sent to the SFB and ARC via two independent transmission chains. The publicly availably industrial power grid is employed to ensure high power supply reliability for all ESSs within the facility. This additionally involves two redundant power supply sources—batteries (AB) with a specified capacity based on the energy balance, and a generator set (GS).

**Figure 5:**
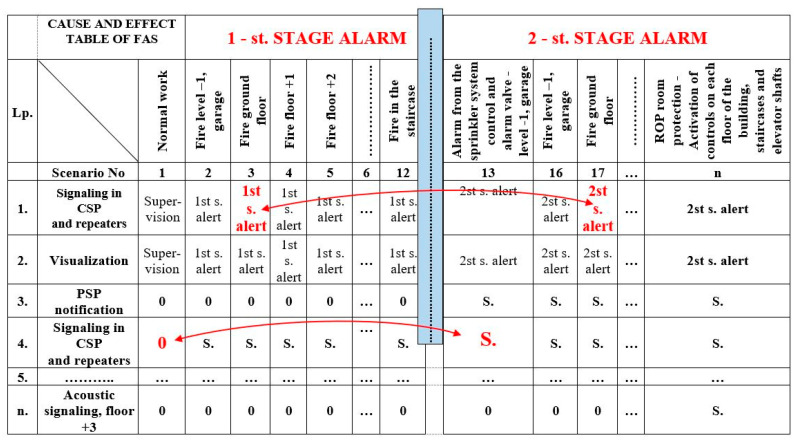
Cause-and-effect table, FAS and GES control matrix, building example as in Figure 4 (arrows in the table indicate passages for two fire alarm cases, stage I and stage II). Key: 0—no controls, S.—fire control, 1st, 2nd stage alarm in FAS.

The organization of the system technical structure, method, and sequence of starting/stopping/triggering/switching, etc., of FAS and GES is a direct result of the fire event scenario and the developed control matrix [9,27,122]. Based on the fire scenario for a given building, the designer takes specific events in the control matrix (Figure 5). This is associated with triggering a given sequence or progression of actions, e.g., two detectors D_12_, D_21_ tripped, room sealing, activation of an electronic control module releasing gas from cylinder BG1, BG2 (valve unlocked after a certain time interval). This includes HVAC deactivation (air inflow isolation), local acoustic and optical signaling system activation, sending alarm signals to SFB and ARC, etc. [3,87,123]. Such is the specific cause-and-effect chain of event sequence consequences within these systems. All electronic, electrical, or mechanical controls should be implemented within this process as reliably as possible [36,124,125]. The reliability and the quality of the system comes about through the efficiency of the designer, who has to take the fire phenomenon into account.

**Figure 6:**
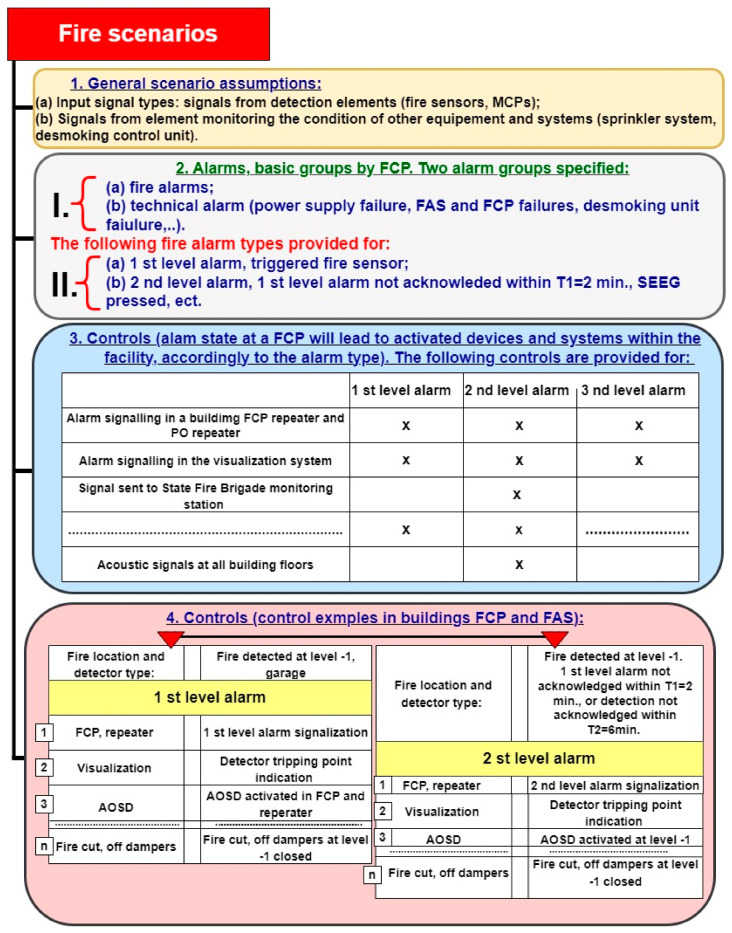
Events occurring within FAS and FCP—fire scenario example (x: technical condition in FAS).

The high burning temperature due to the type of available fire fuel is a particularly adverse external environment factor acting upon individual electronic, mechanical, and electrical elements and devices that make up the entire FAS and GES. Frequent local or widespread fire outbreaks in a given room are difficult to predict when designing such systems [1,16,126]. Such changes not taken into account in the fire scenario (Figure 6) are specific, e.g., during the prolonged operation of a given facility.

This period may, e.g., involve a change in the use of the room or accumulation of various flammable materials. Fire spread in a room or facility is hard to determine and predict [3,9,127]. This leads to an unpredictable FCV spreading in the room. This causes a dynamic change in the natural environment parameters—temperature, smoke, etc. [36,128,129]. Temperature, which particularly impacts FAS and GES elements, causes a dynamic, transient (reversible), or constant (irreversible) change in the FCP and GES reliability structure. Therefore, defining critical paths, i.e., system fitness or unfitness due to fire duration (from the occurrence of this phenomenon until fire is put out by the SFB or the GES), is a crucial issue [1,82,130].

## 4. The Impact of FCV on Elements and Systems Employed within FAS and GES

Environmental conditions, especially temperature and its changes in the event of a fire, are some of the factors that impact reliability (e.g., failure intensity λ) of elements and devices making up specific system structures. A designer of FAS equipment (e.g., sensors, FCP, etc.) always adopts such structural solutions (e.g., selection and arrangement of elements, cooling systems—radiators, etc.), so that they do not exceed the permissible temperature during “normal” operation [36,131,132]. This is particularly important due to the service life, reliability, or functioning of a device employed as part of an ESS. The functioning of FAS and GES devices requires calculating the thermal balance [3,133,134], and such action is associated with comparing the balance of heat generated throughout the operation of a given device with the heat dissipated by the cooling system. The high operating temperature always associated with fire phenomena imparts electrophysical property changes in all electronic subassemblies, both passive and active. These phenomena are [132,135,136]:

- inducing stresses in ESS component structures, e.g., detectors that involve materials with various expansion coefficients, e.g., glass (detector lens), metal (PCB board) or element soldering points (tin), etc.;
- decaying significant stresses due to the functioning of interface materials in the contacts, e.g., detector, assembly socket, NO and NC relay contacts;
- imparting reduced maximum load capacity of conductive materials, e.g., power cables, resistors, or active and passive elements;
- initiating gradual degradation—reducing value and properties of insulating materials, e.g., a temperature increase of 50 °C leads to an approx. 14-fold change in the surface resistivity of the glass–epoxy laminate applied in a PCB.

Based on conducted tests and observations of the electronic equipment operation process, such environmental parameters have significant impact on reliability [36,137,138]. Among others, these include the following:

- Silicon power transistor joint temperature increasing by 10 °C leads to a doubled number of failures. All technical parameters of these elements are subject to change. This includes, e.g., hybrid parameters h_11_ to h_22_ or voltage U_BE_, etc.
- The parameters of passive elements within FCP PCB or detectors are modified. Failure rates double, e.g., in capacitors, upon a temperature increase of 15 °C, in resistors, 35 °C.
- Solder joint strength properties are also changed (reduced twofold) upon a temperature increase from room value (25 °C) to 70 °C.

Ambient temperature affects the operation of entire electronic subassemblies (systems). Therefore, the manufacturers always provide rated and permissible equipment operating values, e.g., the entire heat range within expected use of fire detectors is from −10 °C to + 40 °C, and for ionization smoke (also flame) detectors, it is from −20 °C to +70 °C, with a relative humidity <94% specified for 40 °C. Ensuring rated operating temperature for electronic devices requires the application of forced (e.g., air or water) cooling systems. In the case of FAS or GES, this type of forced temperature stabilization is employed in electronic operation surveillance systems [3,9,139].

Buildings should always take into account a specific fire sequence included in the fire scenario, depending on its location. Many literature publications contain “temperature—time” graphs, which model the courses of certain fire types. The following “temperature—time” waveforms have been defined pursuant to an applicable standard (EN 1363-2:1999) [140]. They simulate the course of fires in rooms according to the standard, hydrocarbon, external, parametric, and tunnel curves. The so-called “temperature–time” standard curve is applied the most often [36,141,142]. Such a curve illustrates so-called ‘cellulose’ fires (where the fuel is mainly wood or wood-like materials) and is commonly applied in studying building fires. The curve can be described by Equation (1).

(1) $$T=345\cdot log\cdot \left(8\cdot t+1\right)\cdot 20$$

where T is temperature, [°C], and t is time, [min].

**Figure 7:**
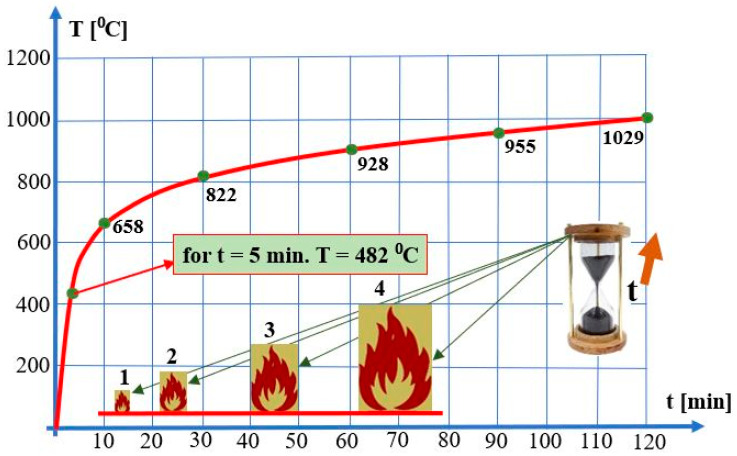
Standard curve—temperature change in cellulose fires.

In the event of a building fire, its temperature approx. 30 min after breakout, reaches approx. 800 °C. The temperature exhibits slight growth tendencies throughout the duration of the fire. T after 60 min ≅ 928 °C, and after t = 90 min, T ≅ 955 °C. Temperature changes due to fire development (cases 1–4) are shown in Figure 7. A temperature increase during a fire entails an increase in the thermal conductivity and a decrease in electrical conductivity (e.g., within conductors supplying FAS and GES elements or devices). In such a case, the conductor resistance for different temperature ranges during a fire can be described by Equations (2) and (3) [3,36,131,132,133,143,144,145].

If the temperature changes up to 200 °C according to the curve, the conductor resistance can be described by a linear relationship, as shown in Equation (2):

(2) $$R_{Tk}=R_{20}\cdot \left(1+\alpha_{20}\cdot \Delta T\right)$$

For temperatures higher than T = 200 °C, the function describing the power conductor resistance becomes nonlinear and is then expressed by relationship (3):

(3) $$R_{Tk}=R_{20}\cdot \left(1+\alpha_{20}\cdot \Delta T+\beta_{20}{\Delta T}^{2}+....\right)$$

where R_20_ is the conductor resistance for a temperature of 20 °C in [Ω], α is the first temperature resistance coefficient at a temperature of 20 °C in [1/K], ΔT = T_k_—20, temperature difference in [°C], T_k_ is the final temperature in [°C], second temperature resistance coefficient at a temperature of 20 °C (relative to the metals employed to construct electrical conductors β_20_ = 10^–6^ K^–2^ expressed in [1/K^2^]). The β_20_ coefficient present in Equation (3) can be determined through expression (4).

(4) $$\beta_{20}=\frac{1}{{2R}_{20}}\cdot \frac{d^{2}\cdot R_{20}}{{dt}^{2}}$$

The increase in the resistance of conductors supplying individual FAS and GES elements during a fire also leads to an additionally increased voltage drop in monitoring lines. All ESS devices consume significantly more supply power at the alarming stage compared to the monitoring stage (monitoring current I_d_ = 0.2 mA; alarming current I_a_—2 mA). For example, for a smoke detector, I_d_ = 0.2 ma, I_a_—5 mA; for an interactive multisensor detector with an acoustic signaling device, I_d_ = 0.2 mA, I_a_—8 mA; for an acoustic and optical signaling device, if the monitoring current is 150 μA, the alarming current is a function of supply voltage, which is as follows: for 12 V (9.6 ÷ 16.0)V ≤ Ia—280 mA, for 24 V (16.0 ÷ 30.0)V, then ≤ I_a_—160 mA. These figures change throughout the entire FAS and GES element operation process, and the significant length of detection loops and lines reaching up to 1000 m from the voltage source (FCP is the supply source) means that the designers must take into account all issues associated with additional voltage drops. This is related to the change in the technical states of these ESSs. These are voltage changes in both the supply lines and the components themselves (e.g., FAS and GES detectors). Operation process models for selected FAS and GES are considered critical paths of in-service performance.

FASs and GESs are the most important ESSs operated in facilities and on sites. These systems have so-called ‘legal basis’ within domestic and international regulations that stipulate their installation locations and methods [146,147]. The legislation, however, lacks clearly specified guidelines on the operational reliability of these systems, particularly during a fire. The operating duration of the systems during a fire is an important issue for people participating in an extinguishing operation. It should be as long as possible, especially when FEDs (e.g., sprinklers, spray nozzles) are operated within the facility. FAS and GES elements, e.g., external trip indicators on an FCP synoptic board, notify people managing the extinguishing operation during a “fire” state as to whether active control or lack of “control” exists rooms or passageways. Therefore, attention needs to be paid to the functional reliability of these systems, during monitoring, alarming, and fire. The “service” life of these devices during a fire should be as long as possible. Hence, a system design should pay attention to the so-called ‘critical paths’ for FAS and GES fitness states during a fire.

### 4.1. Basic Technical Assumptions Regarding FAS and GES Operation Processes Associated with Modeling the Operation of These Systems

The following technical assumptions were adopted in the course of further considerations regarding the operation process of these systems:

- Failure intensity λ for all ESS elements and devices is constant during operation, when there is not a fire, whereas a fire that breaks out causes a change in the failure intensity λ within the operation process. The λ value is always non-negative. All electronic, electric, and electromechanical elements and devices employed in FASs and GESs are subject to a so-called ‘pre-aging process’ at their manufacturing plants. The duration and implementation of this process are always determined at the place of assembly. This most often is a company secret. The execution of this process enables early detection of post-manufacturing failures and defects. It also enables discarding the so-called “infancy” period for failure intensity λ. None of the FASs and GESs employed in buildings are operated until the so-called ‘limit wear moment’. FASs and GESs are (can be) modernized throughout their service life. In certain cases, the modernization process of such systems involves replacement with a new model, with different, better functions (e.g., sensor detection characteristics or sensitivities for fire phenomena) [1,54,148].
- The in-service transition of FASs and GESs to a forced, different technical state, e.g., monitoring–alarm, monitoring–failure, etc., is associated with the implementation of current operational tasks. The current FAS and GES operation process is ahistorical. It is a function that does not depend on the previous operational and functional history. FAS and GES technical state(s) at any time are always only a function that always depends on the state(s) wherein these systems are currently in.

**Figure 8:**
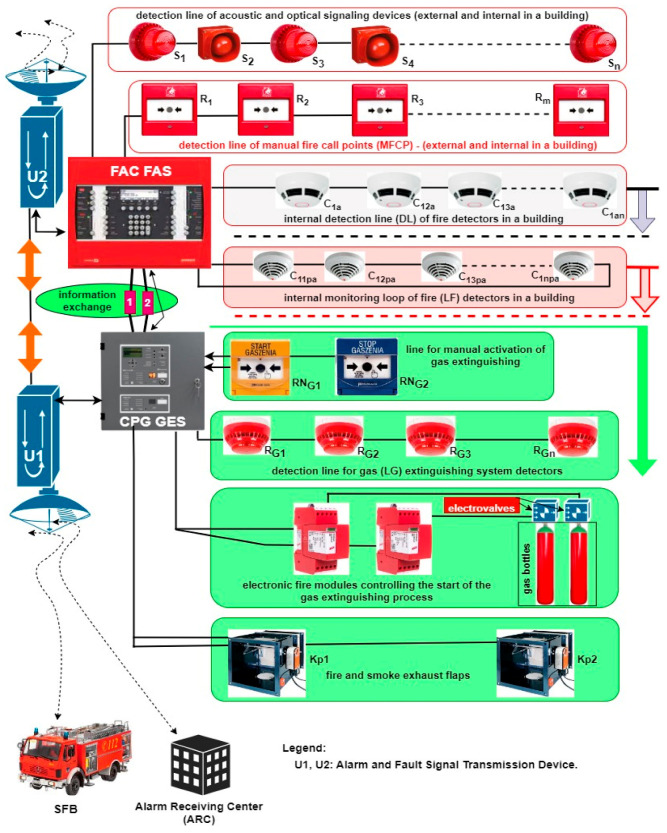
Functional diagram of FAS and GES interaction within a building (key in figure).

Figure 8 illustrates a simplified diagram of FAS and GES operation in buildings. The ESS fitness process is always assessed using diagnostic subsystems that are located within individual alarm control panels (additional, separate modules implementing only these functions in the case of complex systems). The obtained diagnostic information is sent to users operating FASs and GESs, as follows:

- Information sent to the control panel front LCD panel. This is displayed as alphanumeric messages or through LED diode operation, as well as by audio signaling.
- Locally to operational event visualization devices in the ARC. This is usually a computer set-up with a dedicated IT app for operation.
- Fire-only information is always sent to the State Fire Brigade (SFB).

The diagnostic information on the technical state (monitoring, alarm, failure) is sent to ACR and SFB via two telecommunications channels. When analyzing FAS and GES systems, on the basis of the previously mentioned functions illustrated in Figure 8, it can be concluded that it has a mixed-type reliability structure. However, an FCP failure always results in the system shifting from the SPZ state of full fitness to the SB safety unreliability state. Here, FCP unfitness is always critical to the entire FAS. In the case of large, complex, distributed systems, the control panels are always 100% redundant or they follow a known engineering fail-safe reliability principle. Figure 7 does not show the circuit’s primary and back-up power supply systems, and diagnostic and alarm information transmission channels due to figure legibility; however, in the figure, the alarm control panels of the security system exchange information on their technical states via wired links 1, 2 and wirelessly (U1, U2). In the event of a first control panel failure (under the assumptions described in previous article sections), the gas extinguishing process is triggered by the GES CPG (dedicated device) or via FAS FCP by way of an additional extinguishing control module. Information on FAS and GES technical states is transmitted via two independent paths (hard-wired and wireless) to the ARC and SFB. In the case of U1 unfitness, the information on the technical states is forwarded to the aforementioned objects by U2. 

In Figure 8, two detection acoustic and optical signaling device lines (internal and external) are hooked up to the FCP. MFCPs located within passageways automatically trigger the second-level alarm. Moreover, in the figure, a sensor detection loop and the U2 device for transmitting wireless signals have been hooked up to the FCP. Herein, GES is the subsystem monitoring the server room. It is fitted with equipment that enables sealing the room and isolating air-conditioning air supply (K_p1_, K_p2_). Pressure in the cylinders with extinguishing gas is monitored by electronic modules. The cylinders are triggered by solenoids. In the event of non-manual fire detection by the detectors (LG detection line and R_G1_, R_G2_, …, R_Gn_ sensors), the gas is automatically released from the cylinders after a specific period of time intended for the evacuation of people within a given room. It is also possible to manually trigger the entire extinguishing process via RN_G1_. The gas extinguishing process can only be stopped via RN_G2_. Signals related to FAS and GES technical states are encrypted within telecommunication channels and digitally modulated. In the event of unfitness, the gas extinguishing process is automatically triggered from the GES CPG level via the RN_G1_.

### 4.2. Developing Assumptions to the FAS and GES Modeling Process

Taking the aforementioned assumptions into account within the FAS and GES operation process, the authors developed a model wherein two integrated and independently operating fire protection systems have been employed. In the event of GES failure, the operational task can be taken over by the FAS. This is an ordered triple in the form of a relationship described by expression (5).

(5) $$M=\left\langle SB,R,FR\right\rangle$$

where the SB set can be described according to expression (6).

(6) $$SB=\left\{\mathrm{SPZ},\mathrm{SZB},\mathrm{SB}\right\}$$

SB is a set of FAS and GES operational states that can be interpreted as follows:

- SPZ—state of full fitness of integrated FAS and GES; they implement their operational tasks.
- SZB—state of safety hazard No. 1 for FAS and GES (GES unfitness), FAS—FCP takes over the primary role within the gas extinguishing process—first critical path of integrated systems; state of safety hazard No. 2 (FAS unfitness), GES continues to implement operational tasks monitoring the room with electronic devices, but there is the absence of fire protection in the building. The service team immediately takes corrective actions, the second critical path for the operation process.
- SB—state of safety unreliability for ESS–FAS and GES.

States belonging to the SB set can be interpreted as states of full fitness, safety hazard No. 1, safety hazard No. 2, and safety unreliability. This enables considering the technical solutions in terms of FAS and GES operation related to functional safety models.

The second element, RE, found in expression (5) for the ordered M triple is a set of the following pairs with elements interpreted as follows:

- $\left(\mathrm{SPZ},\;\mathrm{SZB}\right)$ is the information on the possible transition of integrated fire ESS from the SPZ state to the SZB1,2 state: FAS, GES—fully fit systems; GES failure—gas extinguishing control taken over by FAS FCP—SZB1; FAS unfitness—fire safety monitored only by GES; SZB2—no fire protection for some building rooms; local service with repair intensity—μ restores full FAS functionality;
- $\left(\mathrm{SZB}1,2;\;\mathrm{SB}\right)$ is the information on the possible transition of integrated fire systems from the SZB1,2 state to the SB state (FAS monitors the entire building, taking over the GES role, while GES monitors the server room). After a specified period of time associated with operation or fire occurrence within the facility, the operated integrated security equipment (FAS and GES) switches to the SB state.

Therefore, the RE element can be described with expression (7).

(7) $$R=\left\{\left(\mathrm{SPZ},\mathrm{SZB}1\right),\left(\mathrm{SPZ},\mathrm{SZB}2\right),\left(\mathrm{SZB},\mathrm{SB}\right),\left(\mathrm{SPZ},\mathrm{SB}\right)\right\},$$

i.e., the RE element can be determined with expression (8).

(8) R⊂S×S

Let us assume that FR is a set of functions, each of which is described using a given RE set. It always adopts values from a set of positive real numbers, i.e., R^+^. The failure intensity function, λ, that characterizes elements, modules, dampers, sensors, and devices employed within FAS and GES takes a specific form. This can be described using expression (9).

(9) $$\lambda :R\underset{\to }{\;}R^{+}$$

Each of the elements in set RE is accordingly assigned a number from set R^+^. This function is interpreted as the intensity of transition within a given operational graph for a given FAS and GES. In particular, the following relationships describing the ESS operation process can be expressed for integrated FAS and GES:

- $\lambda \left(\mathrm{SPZ},\mathrm{SZB}1\right)\equiv \lambda_{1}$ is interpreted as the intensity of a system’s transition from the SPZ state of full fitness to the SZB1 state of safety hazard (GES unfitness, FAS FCP takes over the task associated with the gas extinguishing process for the room where electronic equipment is used (the server room)).
- $\lambda \left(\mathrm{SPZ},\mathrm{SZB}2\right)\equiv \lambda_{12}$ is interpreted as the intensity of a system’s transition from the SPZ state of full fitness to the SZB1 state of safety hazard (FAS FCP unfitness, building fire protection in the server room is implemented only by the GES, local service team takes actions related to FAS recovery, technical state approved by building user). The service team has a strictly specified time to restore system fitness, having an on-site spare parts storage.
- $\lambda \left(\mathrm{SZB}1,\mathrm{SB}\right)\equiv \lambda_{2}$ is interpreted as the intensity of a system’s transition from the SZB1 state of safety hazard to the SB state of safety unreliability (FAS unfitness), with the integrated security system fully unfit.
- $\left(\mathrm{SZB}2,\mathrm{SB}\right)\equiv \lambda_{22}$ is interpreted as the intensity of a system’s transition from the SZB2 state of safety hazard to the SB state of safety unreliability (GES unfitness), with the integrated security system fully unfit.
- $\lambda \left(\mathrm{SPZ},\mathrm{SZ}\right)\equiv \lambda_{11}$ is the intensity of an integrated fire system’s transition from the SPZ state of full fitness to the SB state of safety unreliability, with the system totally unfit. FAS, GES are unfit at time t_0_, e.g., due to intentional electromagnetic interference or, e.g., lightning pulse.

**Figure 9:**
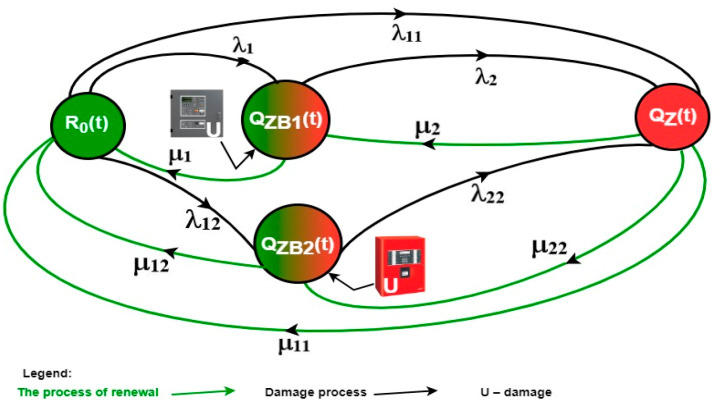
Operation process graph for FAS and GES employed in a building, key in the figure.

A graphical interpretation of the aforementioned operational events related to integrated FASs and GESs that use two independent telecommunication channels and exchange diagnostic information on the technical states via control panels, FAS FCP and GES CPG, is shown in Figure 9. This ensures asymmetrical redundancy, which is the possibility of controlling gas extinguishing also by FAS FCP; however, GES CPG cannot implement fire monitoring (take over the FAS role) of the building’s rooms.

− R_O_(t)—probability function of integrated FAS and GES staying in a state of full fitness; systems implement own operational tasks associated with fire protection.
− Q_ZB1_(t)—probability function for integrated fire safety systems staying in a state of safety hazard 1—GES CPG U failure, system operating in fire monitoring state employing FAS FCP (FCP has an additional, redundant electronic module that enables triggering the entire gas extinguishing procedure in the server room).
− Q_ZB2_(t)—probability function for integrated fire safety systems staying in a state of safety hazard 2—FAS FCP U failure, system operating only in fire monitoring and alarm state employing GES, 24/7 local service team with an on-site spare parts storage required to commence maintenance available in the building. This will enable immediate recovery of an unfit FAS without a time delay, e.g., travel.
− Q_Z_(t)—probability function for integrated FAS and GES staying in a state of safety unreliability. The unfitness of these two systems prevents fire monitoring in the entire building. The recovery process is first implemented in the FAS, followed by the GES. Fire monitoring is implemented by designated (trained) employees operating two systems.

**Figure 10:**
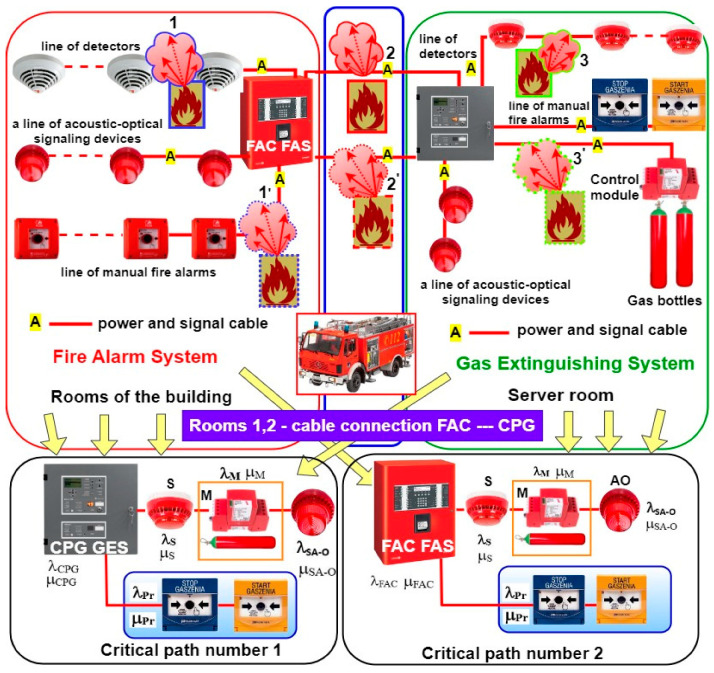
FAS and GES operated in a building; critical paths No. 1 and 2 for the fire scenario and control matrix developed by a designer, where A—power and signal cables, 1.1′; 2.2′; 3.3′—local fire locations (temperature impact on selected elements), failure intensity in elements within critical path No. 1: λ_CPG_ (control panel), λ_S_—detector(s), λ_M_—electronic activation module, λ_SA-O_—acoustic and optical signaling device, λ_Pr_—manual call points (buttons: Start, Stop—gas extinguishing), (λ_FCP_—fire alarm control panel—path No. 2).

Two independent functional reliability critical paths can be determined for the FAS and GES in question. These paths mean satisfying the necessary requirements associated with the occurrence of a fire process. All FAS and GES elements and devices are supplied by a fire-retardant, halogen-free cable, as shown in Figure 10 (designated as A), e.g., of the HLG, HTKSHekw, or HDGS type. Figure 10 shows a simplified action of a so-called ‘local’ (undeveloped) fire on the aforementioned elements of these systems, i.e., cables, components, devices, case from 1.1′, 2.2′, to 3.3′. The temperature generated during a fire has an overall impact on signal and power cables, the routes of which run through given rooms experiencing this phenomenon are shown in Figure 10. A local, undeveloped fire present in a given room generates temperature impact only on the elements (e.g., detectors, modules, etc.) that are confined within a spatially limited area. Because of the presence of extreme temperatures, the connection between two alarm control panels of the system in Figure 10 is via two channels, cables A. The cables must be arranged in two independent rooms, without so-called ‘common’ building partitions, walls, ceilings, or, e.g., doors and windows (usually different fire zones). Figure 10 illustrates two independent system operation functioning paths due to operating reliability. Each critical path ensures the correct functioning and use of GES, according to its intended purpose. 

A fire scenario and control matrix developed by a designer for FAS and GES must take into account the dynamic change in the reliability structure during a fire event. Therefore, it is important in terms of the functional reliability of these systems, especially during a fire, to understand which elements are part of the so-called ‘critical path’. A change in these systems’ critical path reliability structure is dynamic, without any third-party involvement. The parameters of these dynamic changes, time and temperature in particular, are functions dependent on numerous variables, e.g., burned material, i.e., fuel, the temperature in this facility, technical parameters of rooms, such as height, area, construction materials used for walls, ceiling, windows, doors, etc. In addition, this includes air-conditioning (bringing in a constant supply of outside oxygen). In Figure 10, the fire scenario and control matrix, also reflected in the system operation critical paths, employed a manual triggering method (Extinguishing Start and Stop) for GES. In general, GES is activated automatically by detectors within a detection line. The detectors automatically control the M module that opens the solenoid valve and releases gas from the cylinder into the surrounding, limited space (server rooms). GES provides for a manual (operator) method of triggering the aforementioned process. In this case, surveillance is implemented by system operators. In the Figure, each of the elements included in the system operation critical path is characterized by a specific failure intensity, e.g., λ_CPG_ (GES CPG), λ_S_, λ_M_, etc., and recovery intensity μ_CPG_, μ_S_, μ_CPG_, …; FAS FCP can control all GES functions, whereas the GES CPG does not implement any control functions of the second system, i.e., FAS. It is the implementation of the asymmetric redundancy in ESS [2,8,146,147]. 

**Figure 11:**
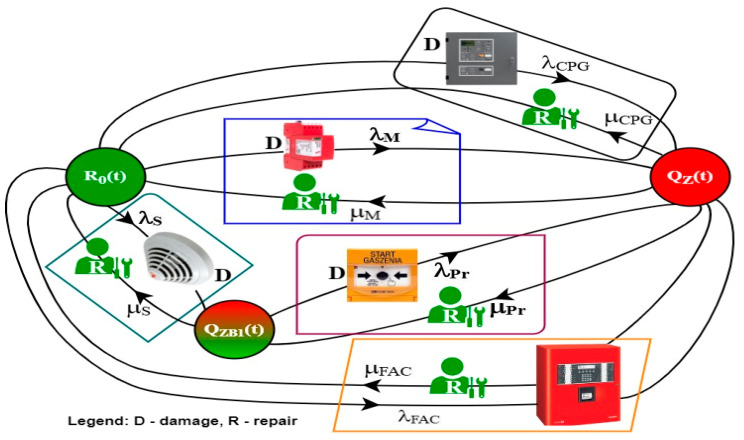
Operation process graph for fire alarm and gas suppression systems (key in figure).

Figure 11 illustrates the operation process graph for an integrated FAS and GES operated in a building. The safety unreliability state (entire system unfitness) Q_Z_(t) can be achieved in the event of CPG—λ_CGP_, FCP—λ_FCP_ or control model λ_M_ failure through a solenoid valve. A simultaneous unfitness of the aforementioned equipment is very unlikely due to the applied technical protections, such as, e.g., short-circuit isolators, varistors, element redundancy, etc. In the case of a failure of sensors λ_S_ located within the detection line hooked up to the CPG (GES automatic operation system), it is possible to manually trigger the GES via the extinguishing start button, as shown in Figure 10.

The fire safety system enables implementing current repairs (local service) through the recovery process, e.g., with an intensity μ_CPG_ (GES control panel)_,_ μ_S_ (detector), μ_FCP_ (FAS control panel), etc. The recovery process is executed immediately after receiving diagnostic information from control panels—FCP and CPG (Figure 10). The designations in Figure 11 are as follows:

− Q_Z_(t)—probability function for the FAS and GES security systems staying in a state of full fitness. Systems implement tasks associated with fire protection.
− Q_ZB1_(t)—probability function for the security systems staying in a state of safety hazard 1; unfitness of detectors connected to the CPG. GES can be triggered via the Gas extinguishing start button.
− Q_Z_(t)—probability function for the fire systems staying in a state of safety unreliability. Transition to the R_0_(t) state means implementing the recovery process.

The model shown in Figure 11 shall be described with the following Kolmogorov–Chapman equations, in order to determine the probabilities of an FAS and GES staying in individual technical states (10). The system of Equation (10) describes the FAS exploitation process for the model presented in Figure 9. For the presented exploitation process model, the authors of the article assumed the so-called ‘pre-ageing’ of all FAS elements, which is carried out at the production plant. In the FAS in question, the devices were manufactured in Poland. The manufacturing plant subjects all components to a pre-aging process for a specified period of time after production before shipping to the manufacturer. This initial process occurring in the production plant makes it possible to assume the further process of FAS operation by the exponential distribution of functioning. After installation, operational tests, and technical acceptance by the user, the FAS is always in a state of complete suitability—R0 (Figure 11). Antidestructive elements and modules are installed in the FAS, e.g., short-circuit isolators, varistors, overvoltage fuses, etc. 

All technical solutions protecting the FAS against interference comply with applicable standards and regulations. However, the occurrence of substantial electromagnetic interference, e.g., in the form of a lightning impulse despite the use of the protections mentioned above, causes the FAS to move to a state of unsuitability (safety failure)—QZ(t) (Figure 11). This is for the case of simultaneous failure of two independent alarm control panels—gas and fire extinguishing control CPG and FAC (markings in Figure 11). This is performed with the damage intensity λ_CPG_ and λ_FAC_, respectively (Figure 11). The use of short-circuit isolators on detection loops in detector and MFCP lines causes the FAS to transition to a state of safety hazard with the intensity of damage λ_s_ and λ_pr_. By undertaking the renewal of these FAS elements, it is possible to achieve Ro status again.

(10) $$\begin{array}{cc} R_{0}^{\prime }\left(t\right)= & -\lambda_{CPG}\cdot R_{0}\left(t\right)+\mu_{CPG}\cdot Q_{Z}\left(t\right)-\lambda_{M}\cdot R_{0}\left(t\right)+\mu_{M}\cdot Q_{Z}\left(t\right)-\lambda_{S}\cdot R_{0}\left(t\right)+\mu_{S}\cdot Q_{ZB1}\left(t\right)-\lambda_{FAC}\cdot R_{0}\left(t\right)\\ & +\mu_{FAC}\cdot Q_{Z}\left(t\right)Q_{ZB1}^{\prime }\left(t\right)=\lambda_{S}\cdot R_{0}\left(t\right)-\mu_{S}\cdot Q_{ZB1}\left(t\right)-\lambda_{Pr}\cdot Q_{ZB1}\left(t\right)+\mu_{Pr}\cdot Q_{Z}\left(t\right)Q_{Z}^{\prime }\left(t\right)\\ & =\lambda_{CPG}\cdot R_{0}\left(t\right)-\mu_{CPG}\cdot Q_{Z}\left(t\right)+\lambda_{M}\cdot R_{0}\left(t\right)-\mu_{M}\cdot Q_{Z}\left(t\right)+\lambda_{Pr}\cdot Q_{ZB1}\left(t\right)-\mu_{Pr}\cdot Q_{Z}\left(t\right)+\lambda_{FAC}\cdot R_{0}\left(t\right)-\mu_{FAC}\cdot Q_{Z}\left(t\right) \end{array}$$

Adopting initial conditions for FAS and GES functioning in the form of expression (11)

(11) $$R_{0}\left(0\right)=1Q_{ZB1}\left(0\right)=Q_{Z}\left(0\right)=0$$

and applying the Laplace transform for the set of Equation (10), we obtain the following set of linear Equation (12):

(12) $$\begin{array}{cc} s\cdot R_{0}\left(s\right)-1= & -\lambda_{CPG}\cdot R_{0}\left(s\right)+\mu_{CPG}\cdot Q_{Z}\left(s\right)-\lambda_{M}\cdot R_{0}\left(s\right)+\mu_{M}\cdot Q_{Z}\left(s\right)-\lambda_{S}\cdot R_{0}\left(s\right)+\mu_{S}\cdot Q_{ZB1}\left(s\right)\\ & -\lambda_{FAC}\cdot R_{0}\left(s\right)+\mu_{FAC}\cdot Q_{Z}\left(s\right)s\cdot Q_{ZB1}\left(s\right)\\ & =\lambda_{S}\cdot R_{0}\left(s\right)-\mu_{S}\cdot Q_{ZB1}\left(s\right)-\lambda_{Pr}\cdot Q_{ZB1}\left(s\right)+\mu_{Pr}\cdot Q_{Z}\left(s\right)s\cdot Q_{Z}\left(s\right)\\ & =\lambda_{CPG}\cdot R_{0}\left(s\right)-\mu_{CPG}\cdot Q_{Z}\left(s\right)+\lambda_{M}\cdot R_{0}\left(s\right)-\mu_{M}\cdot Q_{Z}\left(s\right)+\lambda_{Pr}\cdot Q_{ZB1}\left(s\right)-\mu_{Pr}\cdot Q_{Z}\left(s\right)\\ & +\lambda_{FAC}\cdot R_{0}\left(s\right)-\mu_{FAC}\cdot Q_{Z}\left(s\right) \end{array}$$

Applying inverse transformation to the system of Equation (12), we derive a function form in symbolic terms, expressed by Equations (13)–(15).

(13) $$R_{0}\left(s\right)=\frac{0.322456+3.31046\cdot s+{1\cdot s}^{2}}{0.322524+3.31057\cdot s^{2}+{1\cdot s}^{3}}$$

(14) $$Q_{ZB1}\left(s\right)=\frac{0.000018\cdot \left(0.328622+3.36196\cdot s+1\cdot s^{2}\right)}{\left(0.100768+1\cdot s\right)\cdot \left(0.322524\cdot s+3.31057\cdot s^{2}+1\cdot s^{3}\right)}$$

(15) $$Q_{Z}\left(s\right)=\frac{0.00009215\cdot \left(0.0101316+0.201311\cdot s+1\cdot s^{2}\right)}{\left(0.100768+1\cdot s\right)\cdot \left(0.322524\cdot s+3.31057\cdot s^{2}+{1\cdot s}^{3}\right)}$$

The data on FAS and GES failure intensity λ and recovery intensity μ were obtained through in-service testing for n = 10 different fire safety systems. These systems were operated under the same environmental conditions in specific buildings. All intensity values (failures *λ* and recovery *μ*) for calculations and the conducted computer simulations were calculated as a mean value of the obtained operation process data.

The FAS and GES operation process data adopted for calculating the safety indicators related to these systems’ operation processes are listed in Table 1.

Substituting the data acquired through ESS tests into expressions (13)–(15), we achieve the following function solutions, expressed as Equations (16)–(18).

Ro(s) = μ_Pr_·(λ_Pr_·μ_Pr_ − (s + μ_CPG_ + μ_FCP_ + μ_M_ + μ_Pr_)·(λ_Pr_ + s + μ_S_)))/((−((−λ_CPG_ − λ_FCP_ − λ_M_)·μ_Pr_) + λ_S_·(s + μ_CPG_ + μ_FCP_ + μ_M_ + μ_Pr_)) − (λ_S_·(−μ_CPG_ − μ_FCP_ − μ_M_) − (λ_CPG_ + λ_FCP_ + λ_M_ + λ_S_ + s)·μ_Pr_)·(λ_Pr_·μ_Pr_ − (s + μ_CPG_ + μ_FCP_ + μ_M_ + μ_Pr_)·(λ_Pr_ + s + μ_S_))) (16)

Q_ZB1_(s) = −((−λ_S_·s − λ_S_·μ_CPG_ − λ_S_·μ_FAC_ − λ_S_·μ_M_ − λ_CPG_·μ_Pr_ − λ_FAC_·μ_Pr_ − λ_M_·μ_Pr_ − λ_S_·μ_Pr_)/(s·(λ_CPG_·λ_Pr_ + λ_FAC_·λ_Pr_ + λ_M_·λ_Pr_ + λ_Pr_·λ_S_ + λ_CPG_·s + λ_FAC_·s + λ_M_·s + λ_Pr_·s + λ_S_·s + s^2^ + λ_Pr_·μ_CPG_ + λ_S_·μ_CPG_ + s·μ_FAC_ + λ_Pr_·μ_M_ + λ_S_·μ_M_ + s·μ_M_ + λ_CPG_·μ_Pr_ + λ_FAC_·μ_Pr_ + λ_M_·μ_Pr_ + λ_S_·μ_Pr_ + s·μ_Pr_ + λ_CPG_·μ_S_ + λ_FAC_·μ_S_ + λ_M_·μ_S_ + s·μ_S_ + μ_CPG_·μ_S_ +μ_FAC_·μ_S_ + μ_M_·μ_S_ + μ_Pr_·μ_S_))) (17)

Q_Z_(s) = −((−λ_CPG_·λ_Pr_ − λ_FAC_·λ_Pr_ − λ_M_·λ_Pr_ − λ_Pr_·λ_S_ − λ_CPG_·s − λ_FAC_·s − λ_M_·s − λ_CPG_·μ_S_ − λ_FAC_·μ_S_ − λ_M_·μ_S_)/(s·(λ_CPG_·λ_Pr_ + λ_FAC_·λ_Pr_ + λ_M_·λ_Pr_ + λ_S_·λ_Pr_ + λ_FAC_·s + λ_CPG_·s + λ_M_·s + λ_Pr_·s + λ_S_·s + s^2^ + λ_Pr_·μ_CPG_ + λ_S_·μ_CPG_ + s·μ_CPG_ + λ_Pr_·μ_FAC_ + λ_S_·μ_FAC_ + s·μ_FAC_ + λ_Pr_·μ_M_ + λ_S_·μ_M_ + μ_M_·s + λ_CPG_·μ_Pr_ + λ_FAC_·μ_Pr_ + λ_M_·μ_Pr_ + λ_S_·μ_Pr_ + μ_Pr_·s +λ_CPG_·μ_S_ + λ_FAC_·μ_S_ + λ_M_·μ_S_ + μ_S_·s + μ_FAC_·μ_S_ + μ_CPG_·μ_S_ + μ_M_·μ_S_ + μ_Pr_·μ_S_))) (18)

A solution to the system of Equations (16)–(18) in the time domain can be found in expressions (19)–(21).

R(t) = (0.999801 + 0.0000176834 × 10^−3.21006t^ +0.000180998 × 10^−0.1004725t^) (19)

Q_ZB1_(t) = (0.999801319024147 + 0.000017683438171796346 × 10^−3.21006t^ + 0.000180998 × 10^−0.10047t^) (20)

Q_Z_(t) = (0.9998 +0.00001768 × 10^−3.210065t^ +0.000180998 × 10^−0.10047t^) (21)

### 4.3. Computer Simulation Results for Developed FAS and GES Models

**Figure 12:**
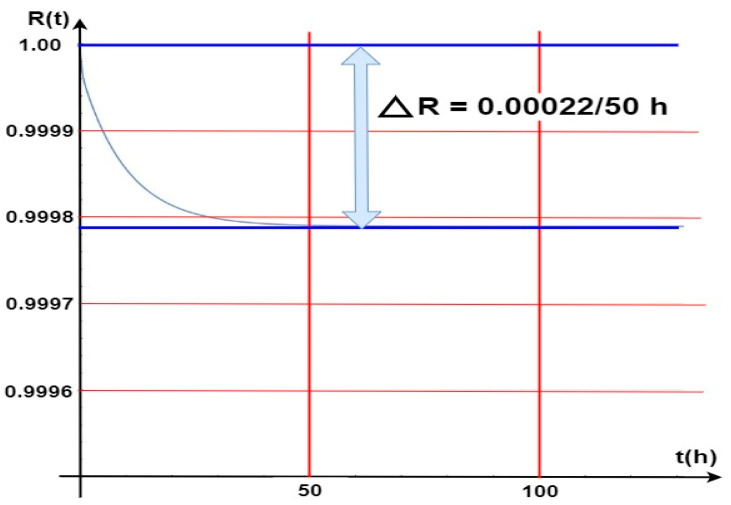
Waveform of the R_0_(t) probability function for all FAS and GES elements and devices staying in a state of full fitness.

Figure 12 shows the waveform of a probability function related to the security systems (FAS and GES) staying in a state of full fitness. In such a case, all security system elements and devices are operational, and power is supplied from an industrial power grid. A battery bank is always treated as a backup power source and is fit (the article does not address issues related to power supply reliability). Diagnostic information on the fitness of all elements and devices is displayed on FCP and GES CPG LCD screens. Such information is developed by a diagnostic subsystem located in two alarm control panels that implement all the functions of electronic security systems on-line. On the basis of the graph in Figure 12, the waveform of the *R(t)* probability function for FAS and GES staying in a state of full fitness (all elements and devices fit), it can be construed that this value was changed very slightly by ΔR(t) = 0.00022 over a very short time, i.e., Δ*t* = 50 [h]. Such a decline in the R(t) function value for the entire fire system comprising GES and FAS is really small, even negligible. In the course of further operation of these security systems, e.g., after t = from 50 to, e.g., 600 h of operation, the R_0_(t*)* probability function for FAS and GES staying in a state of full fitness reaches an approximately constant value equal to R(t) = 0.99978. 

The computer simulation for the aforementioned security systems was conducted for a temperature T = 20 °C, the temperature at which they are operated in the heated/ air-conditioned rooms. Such a high probability of the systems staying in a state of fitness means that the design itself, its practical implementation, and the arrangement and connection of internal and external components, including equipment selection, are correct. FAS and GES elements and devices were selected properly in relation to this system operation process. The environment within which they are operated satisfies all additional assumptions arising from permissible changes in environmental conditions, i.e., permissible operating temperature, changes in humidity, pressure, lighting and electromagnetic interference, etc. 

**Figure 13:**
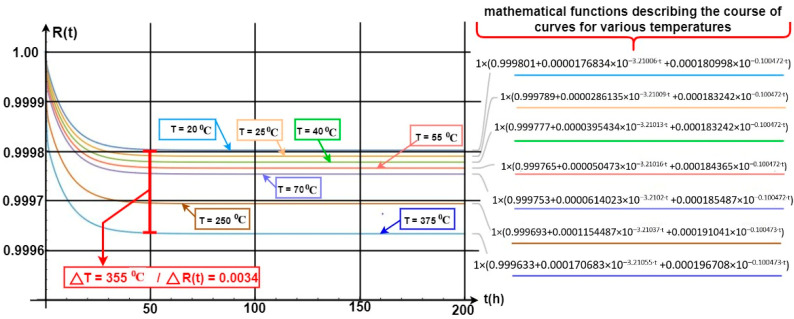
Waveform of the R(t) fitness probability function for FAS and GES in relation to the impact of a local fire on failure intensity λ of module M. Temperature change during a local fire and impact only on a single security system element, module M.

Figure 13 shows the waveforms of the GES and FAS fitness probability function impacted by temperature generated by a local fire in the room. Temperature impacts module M only, as shown in Figure 10, the second security system operation process critical path. Module M is an electromechanical device with a jumper controlled by a gas extinguishing control panel (CSG). Module M is triggered upon sending a current pulse signal from CSG, which opens relief devices releasing pressurized gas into a given room. Oxygen concentration in the monitored room is then decreased (inert gases), and thus, one so-called ‘fire triangle’ reduces to zero after a certain time. This leads to a forced extinguishing process in a room with accumulated computer equipment (server room). Module M is placed in the room in question at a low height above the floor, and is, therefore, exposed to variable, significant temperature, generated during a fire event (GES detectors installed on the ceiling).

The electromechanical devices within the module are resistant to high temperatures, and the signal and power cables enable implementing the extinguishing operation. At the same time, the unfitness of module M is critical for the functioning of both FAS with FCP, and GES. This applies to critical paths 1 and 2, as shown in Figure 10. The temperature in the room reaches simulated values after a certain time, which is a function of numerous variables, e.g., fuel. A temperature increase in a given room by 355 °C leads to a slight decline in the R(t) value by precisely 0.0034. Such a high temperature is important in terms of electronic systems and devices employed in, e.g., detectors, signaling devices, etc. However, these FAS and GES elements are located at a significant distance from the ground (floor), most usually on the ceiling. The temperature in the room does not rise stepwise within a time domain.

**Figure 14:**
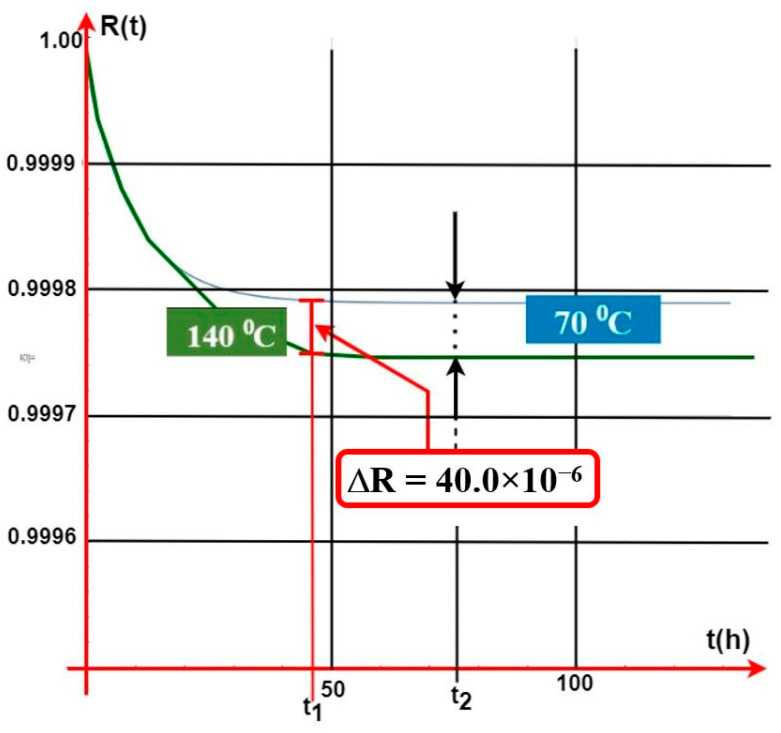
GES R(t) fitness probability function waveform in the case of a temperature change due to a local fire acting upon the M GES module.

Temperature rises according to, e.g., the curve illustrated in Figure 7. R(t) value change functions for individual temperatures impacting module M are also described in Figure 13. Figure 14 illustrates the waveform of the R(t) fitness probability function only for two cases of a change in the temperature acting upon a single GES module M alone. Figure 13 indicates very small changes in the R(t) value at temperature changes ranging from 70 °C to 140 °C. In general, the R(t) fitness probability decreases along with increasing temperature. For the time moment t1 = 47 (h), the change value is only ΔR(t) = 40 × 10−6. After t2 = 75 (h), the R(t) “stabilizes” for these two curves at different, constant levels (values). The R(t) fitness probabilities are at a very high level, ranging from R(t) ≅ 0.9995 to 0.9998 (temperature T = 70 °C). In the case of GES module M, there is a simultaneous, critical failure of two security systems, as shown in Figure 10. The M module is an element critical in ensuring fitness within both the FCP and GES. Because of its location, it is particularly exposed to the impact of high temperature over a fairly short time during a fire. 

## 5. Conclusions

The issues related to the operation process of electronic security systems, FAS and GES in particular, are crucial in buildings where these technical structures are operated, including those classified as state critical infrastructure (e.g., railway and military sites, airfields, logistics bases, etc.). ESSs, which also include FASs and GESs, are always operated within an adverse environment. Therefore, the elements and devices employed to construct them should exhibit high reliability throughout their entire service life. This particularly applies to FAS and GES. The correct selection of elements for technical structures within these systems, taking into account their operational reliability in fire scenarios and control matrices, is particularly imperative. This exceptionally important task must be fulfilled by the designer of these systems, as well as the appropriate services that decide on their implementation in buildings, e.g., SFB. Also, the changes introduced in the course of modernization (replacing elements with others within FAS and GES technical structures) should take operational reliability into account. 

A peculiar problem for the designer is determining fitness critical paths for individual systems, especially at the time of a fire event (e.g., in addition to intrusion). These paths include the so-called ‘critical elements’, the unfitness of which leads to complete FAS or GES failure. Graphs 11 to 13 illustrate the impact of the temperature generated during a fire on two systems—FAS and GES. The impact of temperature was taken into account only for module M, which is a key element within these two fitness paths. The R(t) probabilities of FAS and GES staying in a state of full fitness reach considerable values. A temperature increase in a given room, e.g., by 355 °C, leads to a slight decline in the R(t) value by only 0.0034. Reaching such a high temperature is particularly important for electronic elements, systems, and devices used in, e.g., detectors, signaling devices, etc. However, these systems (FAS and GES) are usually located in the room ceiling (room height), where the temperatures during a fire, local in particular, grow over a long period of time. As shown in Figure 14, very small R(t) value changes take place for the presented temperature variations. For example, the R(t) fitness probability decreases at temperatures ranging from 70 °C to 140 °C. For t_1_ = 47 (h), the change value is ΔR(t) = 40 × 10^−6^, whereas after time t_2_ = 75 (h), the R(t) “stabilizes” for these two curves at different, constant levels (Figure 14). The R(t) fitness probabilities are at a very high level, ranging from R(t) ≅ 0.9995 to 0.9998 at a temperature of T = 70 °C.

In further research related to the impact of temperature and other environmental factors on individual FAS components, the authors of the article plan to use a climatic chamber, and temperature and humidity tests for various manufacturers of FAS components and devices and technical structures will be carried out. Changes in environmental parameters in the climatic chamber will be declared in linear, stepwise, and so-called ‘shock’ (large change, e.g., in temperature, in a short time) scenarios.

## Figures and Tables

**Table 1:** FAS and GES operation process data for calculating safety indicators.

failure intensity of gas suppression control unit λ_CPG_	8.85 × 10^−6^	recovery intensity of gas suppression control unit μ_CPG_	0.5 [1/h]
failure intensity of fire alarm control unit λ_FCP_	12.9 × 10^−6^	recovery intensity of fire alarm control unit μ_FCP_	2.5 [1/h]
failure intensity of module λ_M_	25.2 × 10^−6^	recovery intensity of module μ_M_	0.2 [1/h]
failure intensity of sensor λ_S_	18 × 10^−6^	recovery intensity of sensor μ_S_	0.1 [1/h]
failure intensity of manual extinguishing trigger button λ_Pr_	45.5 × 10^−5^	recovery intensity of manual extinguishing trigger button μ_Pr_	0.01 [1/h]

## Data Availability

The data presented in this study are available on request from the corresponding author.

## Symbols and Abbreviations

ESS	Electronic Security System
FAS	Fire Alarm System
GES	Gas Suppression System
MCP	Manual Call Point
k_g_(t)	availability coefficient
µ	recovery intensity coefficient
λ	failure rate coefficient
R_O_(t)	probability function of an FAS staying in the S_PZ_ state (full fitness)
Q_ZB_(t)	probability function of an FAS staying in the S_ZB_ state (safety hazard)
Q_B_(t)	probability function of an FAS staying in the S_PZ_ state (safety unreliability)
λ	failure intensity, transition of a selected FAS from the S_PZ_ state to the S_ZB_ state
μ	recovery intensity, transition from the S_ZB_ state to the S_PZ_ state
TF1–TF9	test fire designations
P_D1_	detection loop No. 1
λ_CSP_	intensity of transition from the S_PZ_ state of full fitness to the SB state of safety unreliability
SCI	State Critical Infrastructure
FCV	Fire Characteristic Value
ACU	Alarm Control Unit
FCP	Fire Alarm Control Panel
